# L-DOPA in the hu man ovarian follicular fluid acts as an antioxidant factor on granulosa cells

**DOI:** 10.1186/s13048-016-0269-0

**Published:** 2016-09-29

**Authors:** J. Blohberger, T. Buck, D. Berg, U. Berg, L. Kunz, A. Mayerhofer

**Affiliations:** 1 Biomedical Center (BMC), Cell Biology, Anatomy III, Ludwig-Maximilian-University (LMU), Grosshaderner Strasse 9, D-82152 Planegg, Germany; 2 A.R.T. Bogenhausen, D-81675 Munich, Germany; 3 Division of Neurobiology, Department of Biology II, Ludwig-Maximilian-University (LMU), D-82152 Planegg, Germany

**Keywords:** L-DOPA, Granulosa cells, Reactive oxygen species

## Abstract

**Background:**

A previous study showed that dopamine (DA), which is contained in follicular fluid (FF) from IVF patients, strongly increased the production of reactive oxygen species (ROS) by cultured human granulosa cells (GCs). ROS, including H_2_O_2_, are assumed to play roles in ovarian physiology and pathology. Ovarian DA could be derived from the circulation, ovarian innervation and/or unknown ovarian sources. L-DOPA is the direct precursor of DA in its synthetic pathway. It was not yet described in FF. We examined L-DOPA levels in FF from IVF patients. As it may exert anti-oxidative and ROS-scavenging functions, we studied whether it exerts such actions in human GCs and whether DOPA-decarboxylase (DDC), the enzyme converting L-DOPA to DA, is expressed in the human ovary.

**Results:**

ELISA measurements revealed that human IVF-derived FF contains L-DOPA. In cultured human GCs automated confluence analyses showed that L-DOPA enhanced their survival. This is in contrast to the actions of DA, which reduced cell survival. A dose-dependent mode of action of L-DOPA was identified using a fluorescent ROS indicator. The results showed that it antagonized intracellular ROS accumulation induced by exogenous H_2_O_2_. DDC was absent in follicular GCs, but immunohistochemistry identified it in theca cells (TCs) of large follicles in the human ovary. Laser micro-dissection followed by RT-PCR corroborated the expression. DDC was also identified in the steroidogenic cells of the corpus luteum.

**Conclusions:**

L-DOPA in FF is an antioxidant factor and exerts positive influences on GCs. Ovarian DA is derived from L-DOPA and has opposite actions. Exogenous L-DOPA is a standard therapy for Parkinson’s disease, and the results raise the possibility that it may be able to exert positive actions as an antioxidant in ovarian conditions, as well.

**Electronic supplementary material:**

The online version of this article (doi:10.1186/s13048-016-0269-0) contains supplementary material, which is available to authorized users.

## Background

Reactive oxygen species (ROS) are generated in the ovary and are assumed to play roles in ovarian physiology as signaling molecules. They are however also involved in ovarian pathology and act as toxic factors [1, 2].

Previous studies identified the neurotransmitters norepinephrine (NE) and dopamine (DA) in the human ovary [3]. Two studies in cultured human *in vitro* fertilization (IVF)-derived granulosa cells (GCs) showed that NE and DA stimulated the generation of ROS [4, 5] and that the mechanisms involved intracellular uptake and cellular metabolism of the monoamines. The intracellular metabolic breakdown of monoamines generates aldehydes and hydrogen peroxide (H_2_O_2_), which is an important member of the class of ROS.

Monoamine-induced ROS generation is among others linked to the levels of these factors [4, 5] and thus higher levels of DA, for example, resulted in higher ROS levels. Both monoamines were detected in follicular fluid (FF), derived from IVF patients, in substantial levels. When samples from women suffering from PCOS were compared with samples from women without PCOS, a higher concentration of DA was apparent [4]. While NE is presumably mainly derived from the circulation and sympathetic fibers innervating the wall of follicles, the reason for the high concentrations of DA in the human ovary and specifically in PCOS patients remains unknown. It raises the question of local production in the ovary. DA is synthesized in several steps starting with the ubiquitously available amino acid L-tyrosine. Previous studies failed to reveal the presence of the rate-limiting enzyme tyrosine-hydroxylase (TH) other than in ovarian nerves [6, 7]. Thus a substantial *de novo* synthesis in the ovary is unlikely.

DA can be generated from L-DOPA by dopamine decarboxylase (DDC), an enzyme expressed in many cells of the body. Generation of DA from exogenous L-DOPA is the basis for the standard treatment of Parkinson's disease patients [8–10]. Interestingly, DA likely contributes to neuronal cell death in Parkinson's disease by its ability to increase ROS [11]. In contrast, its precursor L-DOPA has antagonist actions and lowers ROS in neuronal cells [12]. The mechanisms of actions are not fully explored, but they include induction of cellular response, as detailed in recent studies [12, 13]. These include changes in metabolic routes, cytoskeletal integrity, pCREB and CD39 expression, to name a few. In addition, ROS scavenging abilities of L-DOPA have been established, as well [14].

The presence of high amounts of DA in FF and the lack of knowledge about L-DOPA in FF prompted us to explore these points. We examined human FF, GCs and ovarian sections.

## Methods

### Human GC isolation, culture and treatment

As described previously, human GCs were derived from FF aspirates of IVF patients stimulated according to routine protocols, in general the “long” stimulation protocol was used [4, 5, 15–18]. The patients underwent IVF treatment primarily for male factor infertility. Age varied and ranged up to 39 years. In addition, from some of the IVF-patients blood samples were drawn and serum was kept at −20 °C for later analyses. FF aspirates from two to five patients were pooled for GCs preparation, following a method previously described [19]. It involves a cell strainer (40 μm; BD, Franklin Lakes, NJ, USA) for filtration of the aspirates. GCs, which remained in cell strainer, were retrieved by washing with Dulbecco’s modified Eagle’s medium (DMEM)/Ham’s F12 Medium (Gibco – Life Technologies, Carlsbad, CA, USA). The filtrate was centrifuged and the supernatant (i.e., cell-free FF) was frozen at −20 °C until further use. Remaining cell aggregates in the acquired cell suspension were suspended mechanically by using a 0.9-mm cannula. Washed cells were re-suspended and cultured in DMEM/Ham’s F12 medium supplemented with penicillin (100 U/ml), streptomycin (100 μg/ml) and 10 % FCS (all from PAA) [15–18]. Primary GCs were cultured for up to 6 days. Cells were rinsed on day 1 of culture with fresh medium to remove non-adherent and dead cells. For all experiments, DMEM/Ham’s F12 medium without supplements was used. DA (Sigma-Aldrich, St Louis, MO, USA) and L-DOPA (Sigma-Aldrich) were used in several experiments.

### Confluence measurement

GCs were cultured up to 4 days and confluence of GCs was monitored for 24 h by taking time-lapse pictures every 10 min using a live cell analyzer (Peqlab, Erlangen, Germany). For each stimulation protocol (DA 200 nM, L-DOPA 200 nM), respective control cells were observed simultaneously. The software of the live cell analyzer determined confluence values.

### ELISA measurements of L-DOPA

L-DOPA was determined in FF and serum using an enzyme-linked immunosorbent assay (ELISA) kit (Bioassay Technology Laboratory, Shanghai, China), following the instructions of the manufacturer. According to this information, the assay was validated for human serum, body fluids, tissue and cell culture media. Measurements were performed in microtiter plates, and reactions were monitored at 450 nm in a microplate reader (BMG labtech, Ortenberg, Germany). L-DOPA in the range of 5–3200 pg/ml can be detected with this assay. The samples were diluted 1/20 for measurements.

### Measurement of ROS

The 2-,7-dichlorodihydrofluoresceindiacetate (DCFH_2_-DA) method was used, as described [4, 5]. In the presence of intracellular ROS, such as H_2_O_2_, the DCFH_2_-DA dye (Invitrogen, Karlsruhe, Germany), after intracellular ester hydrolysis, is converted to the highly fluorescent compound 2-,7-dichlorofluorescein (DCF). For quantification of ROS, GCs were placed in 96-well plates (Thermo Fisher Scientific, Waltham, MA, USA) and loaded with DCFH_2_-DA (10 mM) for 30 min at 37 °C in DMEM/Ham’s F12 medium without phenol red (Gibco - Life Technologies). Fluorescence levels were measured at 485 nm excitation/520 nm emission in a microplate reader (BMG labtech) during stimulation with L-DOPA (200 nM) and H_2_O_2_ (1 mM). After 2 h, when the experiments were terminated, the final values were statistically analyzed.

### Measurement of H_2_O_2_ generation

H_2_O_2_ was measured by using an Amplex Red kit (Invitrogen - Life Technologies, Carlsbad, CA, USA) following the instructions of the manufacturer. Amplex Red was used in a final concentration of 2.5 μM. GCs were placed in 96-well plates and fluorescence levels were measured for 2 h at 544 nm excitation/590 nm emission in a microplate reader (BMG labtech). The blank for each value was subtracted.

### Immunohistochemistry

Sections of human ovaries derived from a local collection at Anatomy III, Cell Biology (Munich, Germany) were used for immunohistochemistry. Immunohistochemistry was performed with a rabbit antiserum raised against human DDC (Sigma-Aldrich) and the corresponding blocking peptide (Sigma-Aldrich). Tissue samples and immunohistochemistry were described previously [5, 18]. In brief, after removal of paraffin, antigen retrieval and blocking of endogenous peroxidase activity, the tissue was incubated in 5 % appropriate serum, diluted in phosphate-buffered saline. Antiserum incubation was done overnight at 4 °C. The antiserum against human DDC was diluted 1:50. Afterwards, incubation with a biotinylated secondary antibody (1:500 dilution; Dianova, Hamburg, Germany) for 2 h at room temperature was performed. A Vectastain ABC Kit (Vector Laboratories, Burlingame, CA, USA) and a 3,3′-diaminobenzidine tablet set (Sigma-Aldrich) were used for the final staining procedure. Slides were covered with Entellan (Merck Millipore, Billerica, MA, USA). For control purposes, incubation with antigen peptide pre-adsorbed antiserum was employed.

### Laser microdissection

Human ovarian tissue samples embedded in paraffin were cut into sections and mounted on a polyethylene naphthalene membrane, as previously described [20]. For dissection of the cellular compartments a laser microdissection (LMD) device (P.A.L.M. Microlaser Technologies, Bernried, Germany) was used. RNA isolation was performed by using a RNeasy Mini Kit (Qiagen, Hilden, Germany).

### Reverse transcription-PCR

Total RNA of cultured cells was isolated using the RNeasy Mini Kit (Qiagen, Hilden, Germany). Reverse transcription was performed with 400 ng RNA using random hexamer primers and Superscript II (Life Technologies). Human liver cDNA (Clontech, Mountain View, CA, USA) was used as positive control. RT-PCR (semi-nested) was arranged with different oligomer primers (DDC forward: 5-GCCCCTACTTCTTCGCCTAC-3; DDC forward nested: 5-ATGCTTGCGGACATGCTGTG-3; DDC reverse: 5-CAGTCTCCAGCTCTGTGCAT-3). PCR products were analyzed by using agarose gel electrophoresis with Midori Green (Nippon Genetics Europe GmbH, Düren, Germany) staining. All products were confirmed by sequencing [5].

### Statistics

Statistical analyses were done using Prism 5 (GraphPad Software, San Diego, CA, USA). For the confluence measurements an unpaired *t*-test was performed (*P* < 0.05). A one-way ANOVA followed by the Newman–Keuls post-test (*P* < 0.05) was performed for ROS (DCFH_2_-DA) measurements.

## Results

**Fig. 1:**
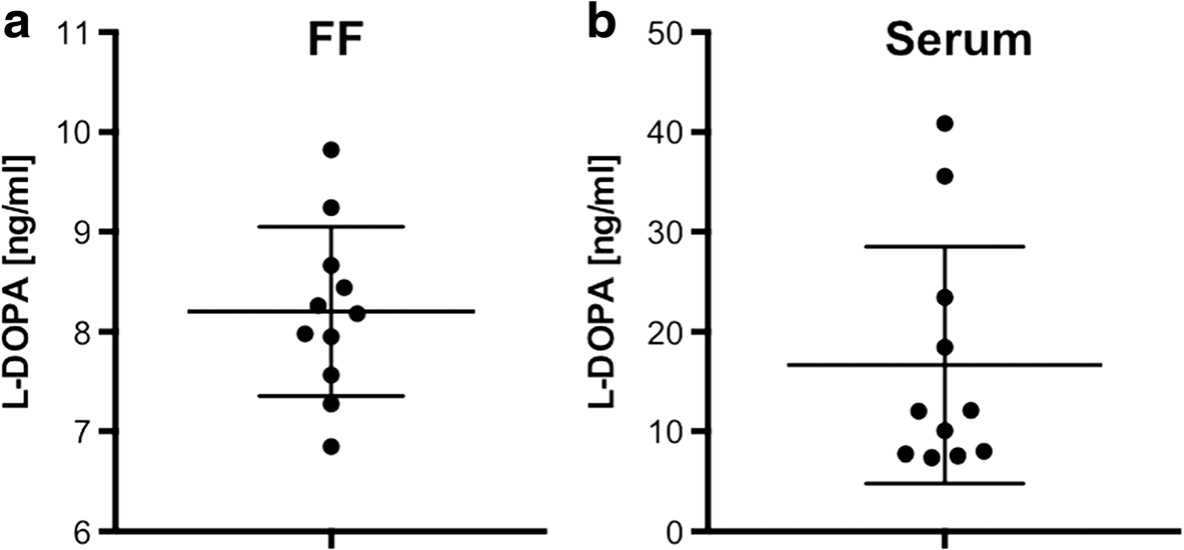
L-DOPA levels in follicular fluid and serum. **a** L-DOPA levels in in vitro fertilization (IVF)-derived follicular fluid samples. Individual values of 11 FF are given, as well as the mean and SD. **b** Individual values of L-DOPA in sera from 11 patients are given, as well the mean and SD. Note that the samples shown in **a** and **b** do not stem from the same patients

We detected L-DOPA in IVF-derived FF by using ELISA measurements (Fig. 1a; *n* = 11 preparations). The levels varied (range 6.8–9.8 ng/ml), but on average a concentration of 8.2 ng/ml was quantified. L-DOPA was also detected in serum samples, obtained from a total of 11 different women (Fig. 1b). The levels varied and ranged from 7.4 to 40.9 ng/ml, with an average of 16.7 ng/ml.

**Fig. 2:**
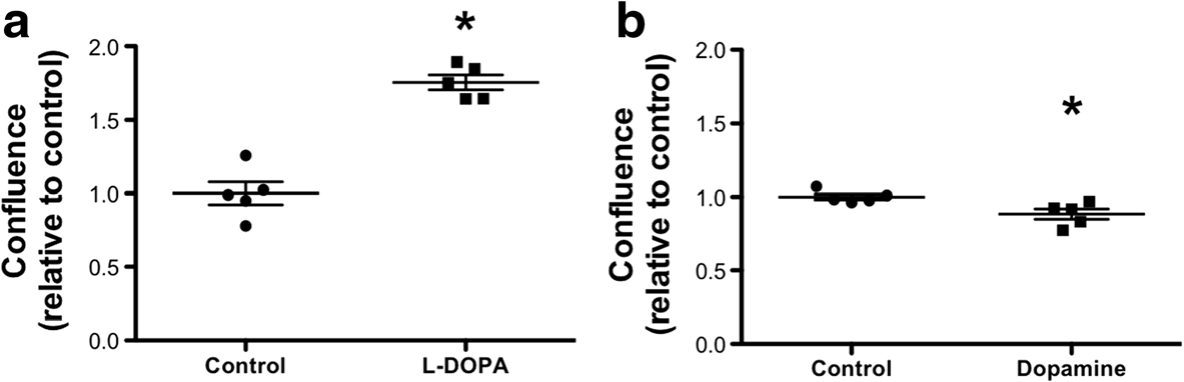
DA and L-DOPA effects on cultured human GCs. **a** Treatment with L-DOPA (200 nM) causes a significant increase of confluence in human GCs after 24 h compared to control group (*P* < 0.05, *t*-test). **b** DA (200 nM) stimulation decreases confluence in human GCs after 24 h compared to control group (*P* < 0.05, *t*-test). All values are shown as well as mean ± S.E.M. of *n* = 5 independent preparations of cells from two to five patients each

Next, the role of L-DOPA was explored in cultured human GCs. L-DOPA in the medium enhanced the survival of cultured GCs (*P* < 0.05). This is concluded from automated confluence measurements of cultured GCs, treated with L-DOPA compared to control cells (Fig. 2a). When DA was used instead of L-DOPA, confluence was reduced (*P* < 0.05) indicating reduced cell survival (Fig. 2b).

**Fig. 3:**
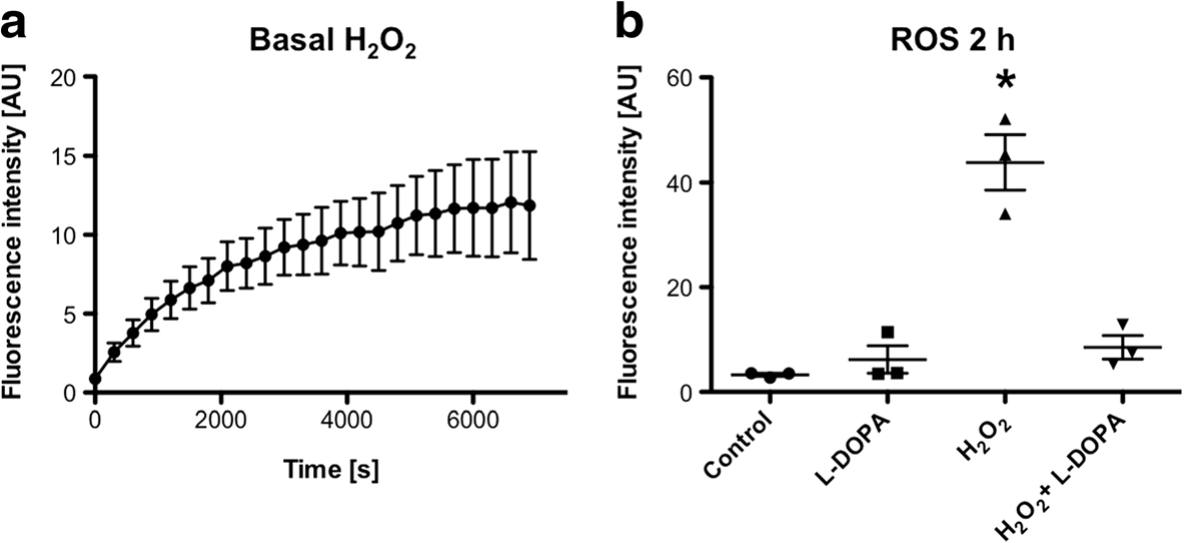
Production of H_2_O_2_ and ROS in cultured human GCs. **a** Measurement of H_2_O_2_ using amplex *red* shows generation of H_2_O_2_ occurring over 2 h. Values are shown as mean ± S.E.M of *n* = 3 experiments. **b** L-DOPA (200 nM) significantly blocks H_2_O_2_ (1 mM) dependent DCF fluorescence intensity (*P* < 0.05, ANOVA, Newman-Keuls). Values are shown as mean ± S.E.M. of *n* = 3 independent preparations of cells from two to five patients each. Note that shown values are resulting from endpoint measurements after 2 h

Amplex red assays revealed that H_2_O_2_ is a specific product of cultured human GCs under basal conditions. H_2_O_2_ levels increased during a 2 h measurement. This was seen in several independent measurements (Fig. 3a). We speculated that the trophic role of L-DOPA may be related to an interference with H_2_O_2_ and or its consequences in GCs. This mode of action of L-DOPA was identified in further experiments using the ROS indicator DCF and exogenous H_2_O_2_. H_2_O_2_-scavenging actions of L-DOPA were observed, which occurred rapidly and were depending on the concentration of L-DOPA (Fig. 3b; Additional file 1). Concentration levels as low as 20 nM were sufficient for a significant effect.

**Fig. 4:**
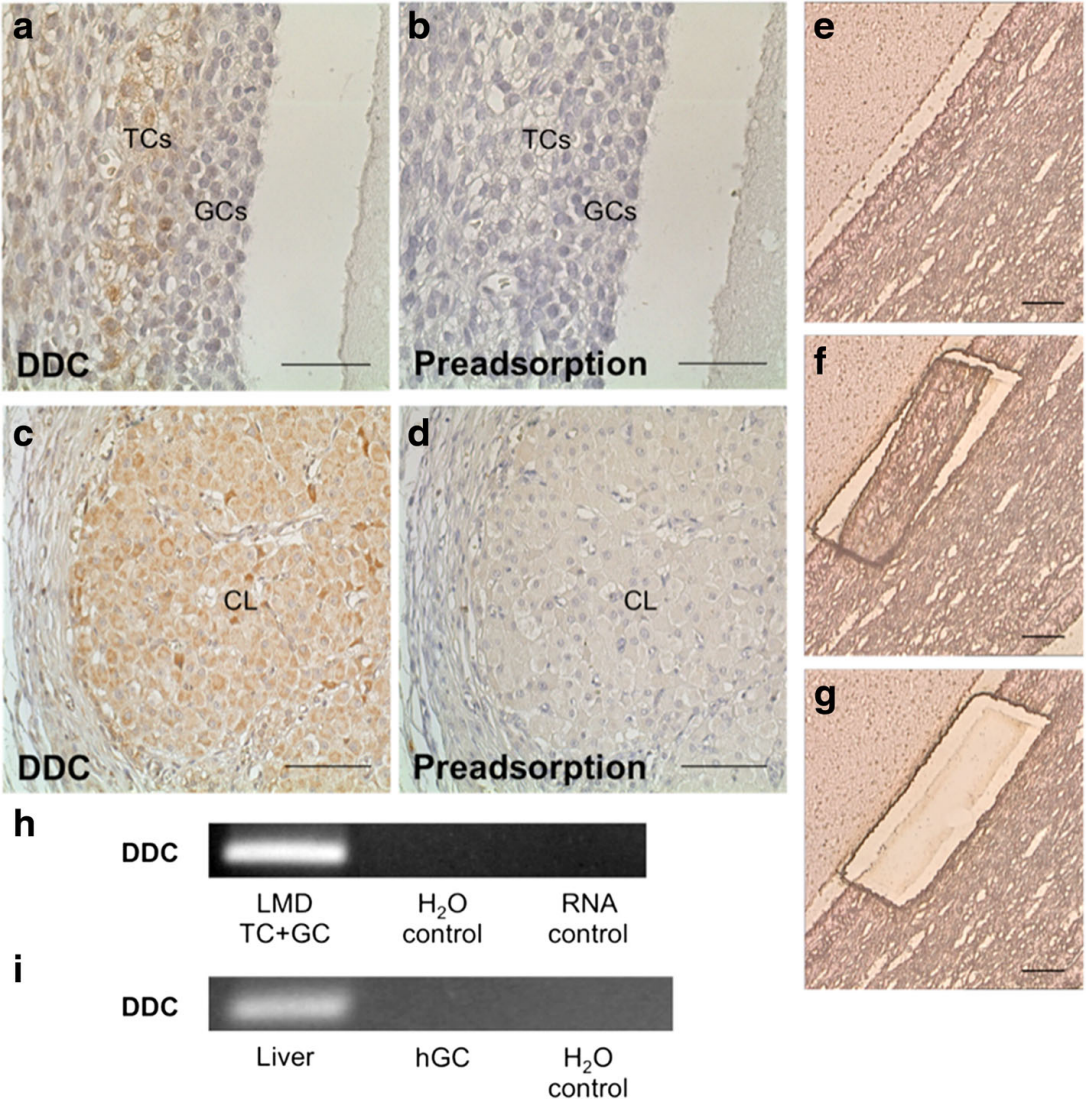
DDC in human ovarian tissue. **a** In human ovarian tissue TCs are positive for DDC in an immunohistochemical staining. **b** The pre-adsorption control is devoid of staining. **c** Cells of the human corpus luteum are positive for DDC using immunohistochemistry. **d** Pre-adsorption abolished staining of the corpus luteum. **e**–**g** Micrographs of human ovarian tissue before (**e**), during (**f**) and after (**g**) LMD. TCs and GCs of the follicle wall were excised. After RNA extraction a RT-PCR was performed. Bars indicate 50 μm (**a** and **b**) and 100 μm (**c**–**g**). **h** RT-PCR and sequencing showed, that DDC mRNA is present in samples of human TCs/GCs. All controls (input of H_2_O instead of cDNA and RNA instead of cDNA) were negative. **i** DDC mRNA is absent in cultured human GCs (hGC; pool of seven preparations). Human liver cDNA was used as positive control. Controls (input of H_2_O instead of cDNA) were negative

DDC converts L-DOPA to DA and immunohistochemistry showed its presence in the theca cells (TCs) but not GCs of large human follicles and the cells of the CL in the human ovary. The pre-adsorption control supports specificity of the immunohistochemical staining (Fig. 4a-d). Results of laser microdissection studies of the cells of the follicular wall followed by RT-PCR (Fig. 4e-h) also showed the presence of DDC in this tissue. In cultured human GCs, DDC mRNA was not detectable (Fig. 4i). DA was reported previously in FF in higher concentrations than in serum ([4]), suggesting a local synthesis.

## Discussion

To our knowledge neither presence nor functions of L-DOPA in the human ovary, in FF and human GCs have been examined. We readily detected L-DOPA in IVF-derived FF and found that the concentration range is somewhat lower compared to serum. Serum values of L-DOPA in women undergoing IVF are not published to our knowledge, but the reported serum/plasma levels in humans vary substantially, when determined by HPLC [21]. In comparison, our results obtained by ELISA measurements indicate somewhat higher levels than described [22]. This observation may be conditioned by the IVF treatment, since L-DOPA may to be linked to gonadotropin levels [23, 24], but also be due to inter-individual variation due to metabolism and nutrition. Thus further studies will require extensive recruitment of patients.

L-DOPA has anti-oxidative abilities [12, 13]. The mechanisms are not fully known, but include a ROS scavenger action, as well as induction of gene expression. The nature of L-DOPA to specifically antagonize H_2_O_2_ was previously shown [12, 14, 25]. In GCs the rapid onset of effects, which were observed in the ROS measurements within minutes (Additional file 1), support the scavenger abilities of L-DOPA. Other actions of L-DOPA in GCs remain to be shown.

We found that GCs produce H_2_O_2_, but the reasons remain to be fully elucidated. Several enzymes, namely steroidogenic enzymes, oxidases (specifically the NOX 4 and 5 enzymes [26]) may be responsible for ROS generation.

DA, formed by DCC from L-DOPA, is a factor described previously in high concentrations in FF and it increases the generation of H_2_O_2_ in GCs. This is due to uptake and cellular metabolism of DA [4]. In GCs this may lead to oxidative stress and our studies using an automated evaluation of cell confluence indeed revealed a deleterious action of DA. In contrast L-DOPA increased cell confluence. Cell confluence is a measure for cell viability, cell number and/or size and increases seen in the L-DOPA group can be interpreted as overall trophic action [4, 5, 26]. Results of cell counting after exposure to L-DOPA confirm this assumption (data not shown). Clearly, a detailed evaluation of the cellular events, which may also include cell death events, are required to decipher the actions of L-DOPA.

If transferable to the in vivo situation, DA and L-DOPA thus may act antagonistically on the ROS environment of the follicle. The present study also indicates that L-DOPA levels in FF are somewhat lower than the ones in serum, at least in samples available to us. However, FF and serum samples do not stem from the same women, hence this point must be evaluated further.

The samples from human CLs available to us were positive for DDC protein. They likely stem from the mid phase of the life span of the CL. Unfortunately the expression of DDC in other phases of the CL (formation, regression) could not be studied due to a lack of samples. We did not detect DDC in IVF-derived cultured GCs. They were cultured under basal conditions for a few days and may represent cells comparable to the young, forming CL. This may in part explain the discrepancy but, clearly, a more detailed study on DDC-expression during the life span of the CL will be required.

Expression of DDC in TCs and in the CL imply that DA can be generated in the ovary from its immediate precursor, rather than from L-tyrosine, since TH, the rate limiting enzyme, was reported in neuronal elements but not in TCs and GCs of the human ovary [7]. In brain expression of different catecholamine-synthesizing enzymes in different neurons were reported [27], implying that synthesis of catecholamines not necessarily depends on *de novo* synthesis from L-tyrosine in all cells.

With regard to the ovarian pathophysiology, DCC expression in TCs may imply that their number and the presence of a CL could be correlated with higher DA levels within the ovary. The relevance for PCOS, in which hyperthecosis is reported, remains to be shown. However we have previously shown higher DA in FF of PCOS women undergoing IVF [4], a result that could be related to more TCs (hyperthecosis) expressing DDC.

In summary, L-DOPA could be an antioxidant factor of relevance for the human ovary. This conclusion from our studies raises the question whether an enhanced exogenous supply of L-DOPA may be a novel therapeutic approach to treat ovarian conditions related to oxidative stress. Furthermore, to our knowledge positive actions of systemic L-DOPA in mice were reported. They include a small increase in fertility, namely increased litter size [28]. Reports in human are however to our knowledge almost missing, a fact related to the onset of Parkinson's disease after childbearing years [29]. Only one brief study stemming from 1980 reported that L-DOPA positively affects female fertility [30].

## Conclusions

Taken together our results indicate that L-DOPA is an antioxidant factor in the human ovarian system. In contrast to DA it shows positive effects on granulosa cells. Since L-DOPA is used in therapy of Parkinson disease, it may be relevant for treatment of ovarian diseases as well.

## Additional file

Additional file 1: ROS generation in cultured human GCs. **A** L-DOPA (2 nM, 20 nM) reduces H_2_O_2_-dependent (1 mM) DCF fluorescence intensity during 2 h of stimulation. **B** Endpoint measurements of cells treated witch L-DOPA (2 nM) after 2 h show a slightly reduced H_2_O_2_-dependent ROS signal. A L-DOPA concentration of 20 nM leads to a significantly decreased ROS generation (*P* < 0.05, ANOVA, Newman-Keuls). All values are shown as mean ± S.E.M. of *n* = 4 independent preparations of cells from two to five patients each. Different letters indicate statistically significant differences between the treatment groups. (DOCX 192 kb)

## Abbreviations

DA: Dopamine
DCFH2-DA: 2-,7-dichlorodihydrofluoresceindiacetate
DDC: 3,4-dihydroxyphenylalanine decarboxylase
DMEM: Dulbecco’s modified Eagle’s medium
ELISA: Enzyme-linked immunosorbent assay
FF: Follicular fluid
GC: Granulosa cell
H_2_O_2_: Hydrogen peroxide
IVF: *In vitro* fertilization
L-DOPA: L-3,4-dihydroxyphenylalanine
LMD: Laser microdissection
LMU: Ludwig-Maximilian-University
NE: Norepinephrine
ROS: Reactive oxygen species
RT-PCR: Reverse transcription PCR
TC: Theca cell
TH: Tyrosine-hydroxylase
