# Supplementary material for: L-DOPA in the hu man ovarian follicular fluid acts as an antioxidant factor on granulosa cells
Source: J Ovarian Res. 2016 Sep 29;9:62. doi: 10.1186/s13048-016-0269-0 (PMC5043631; doi:10.1186/s13048-016-0269-0)
Supplement: Additional file 1: — ROS generation in cultured human GCs. A L-DOPA (2 nM, 20 nM) reduces H2O2-dependent (1 mM) DCF fluorescence intensity during 2 h of stimulation. B Endpoint measurements of cells treated witch L-DOPA (2 nM) after 2 h show a slightly reduced H2O2-dependent ROS signal. A L-DOPA concentration of 20 nM leads to a significantly decreased ROS generation (P < 0.05, ANOVA, Newman-Keuls). All values are shown as mean ± S.E.M. of n = 4 independent preparations of cells from two to five patients each. Different letters indicate statistically significant differences between the treatment groups. (DOCX 192 kb) [file 13048_2016_269_MOESM1_ESM.docx]

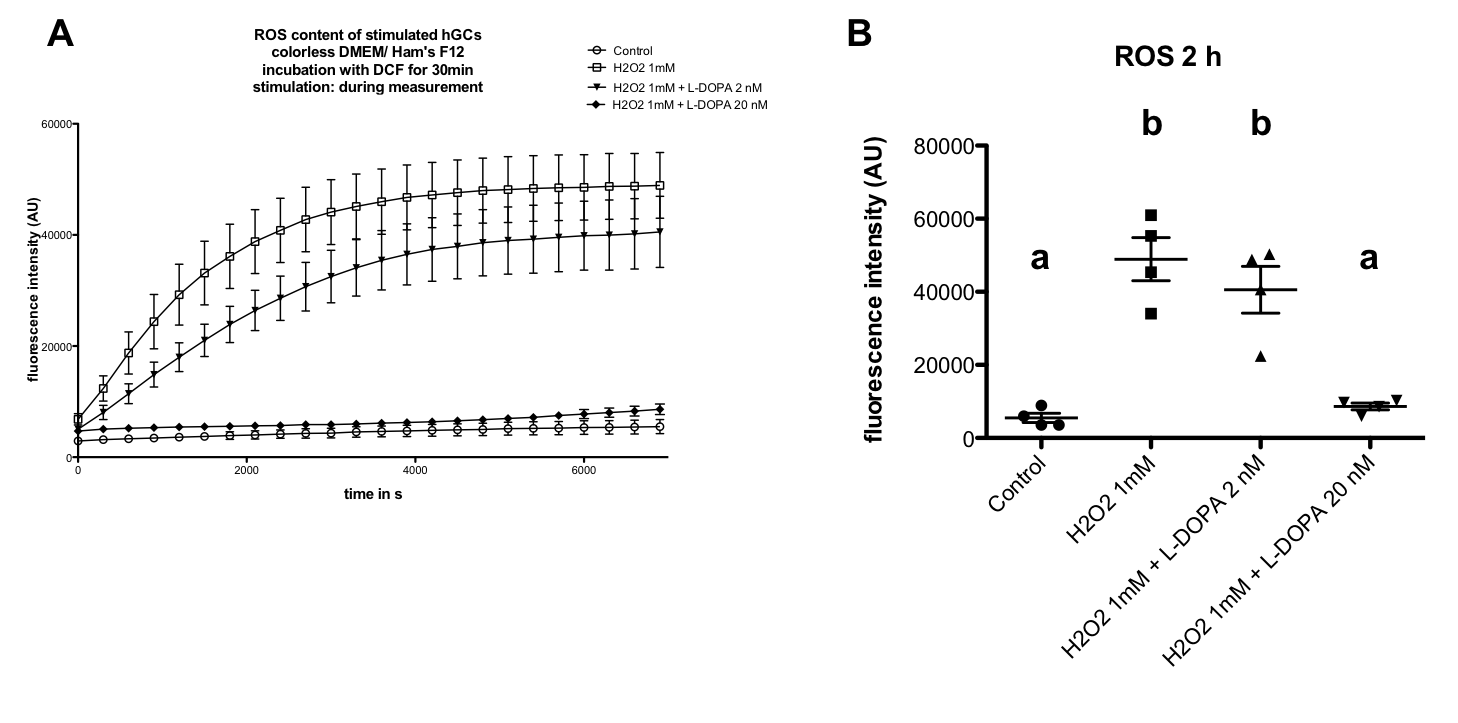


**Additional file 1.** ROS generation in cultured human GCs. **A** L-DOPA (2 nM, 20 nM) reduces H_2_O_2_-dependent (1mM) ROS production during 2 h of stimulation shown by DCF fluorescence intensity. **B** Endpoint measurements of cells treated witch L-DOPA (2 nM) after 2 h show a slightly reduced H_2_O_2_-dependent ROS signal. A L-DOPA concentration of 20 nM leads to a significantly decreased ROS generation (*P* < 0.05, ANOVA, Newman-Keuls). All values are shown as mean ± S.E.M. of n = 4 independent preparations of cells from two to five patients each. Different letters indicate statistically significant differences between the treatment groups.
